# Tracking Cavity Formation in Electron Solvation: Insights from X-ray Spectroscopy and Theory

**DOI:** 10.1021/jacs.3c11857

**Published:** 2024-01-25

**Authors:** Arturo Sopena Moros, Shuai Li, Kai Li, Gilles Doumy, Stephen H. Southworth, Christopher Otolski, Richard D. Schaller, Yoshiaki Kumagai, Jan-Erik Rubensson, Marc Simon, Georgi Dakovski, Kristjan Kunnus, Joseph S. Robinson, Christina Y. Hampton, David J. Hoffman, Jake Koralek, Zhi-Heng Loh, Robin Santra, Ludger Inhester, Linda Young

**Affiliations:** † Center for Free-Electron Laser Science CFEL, Deutsches Elektronen-Synchrotron DESY, Notkestraße 85, Hamburg 22607, Germany; ‡ Chemical Sciences and Engineering Division, Argonne National Laboratory, Lemont, Illinois 60439, United States; § Department of Physics and James Franck Institute, The University of Chicago, Chicago, Illinois 60637, United States; ∥ Center for Nanoscale Materials, Argonne National Laboratory, Lemont, Illinous 60439, United States; ⊥ Department of Applied Physics, Tokyo University of Agriculture and Technology, Tokyo 184-8588, Japan; # Department of Physics and Astronomy, Uppsala University, Box 516, Uppsala SE-75120, Sweden; ¶ Laboratoire de Chimie Physique-Matière et Rayonnement, LCPMR, Sorbonne Université, CNRS, Paris F-75005, France; ∇ LCLS, SLAC, Menlo Park, California 94025, United States; ○ School of Chemistry, Chemical Engineering and Biotechnology, and School of Physical and Mathematical Sciences, Nanyang Technological University, Singapore 637371, Singapore; ⧫ Department of Physics, Universität Hamburg, Notkestraße 9, Hamburg 22607, Germany; △ Department of Chemistry, Northwestern University, 2145 N. Sheridan Rd., Evanston, Illinois 60208, United States

## Abstract

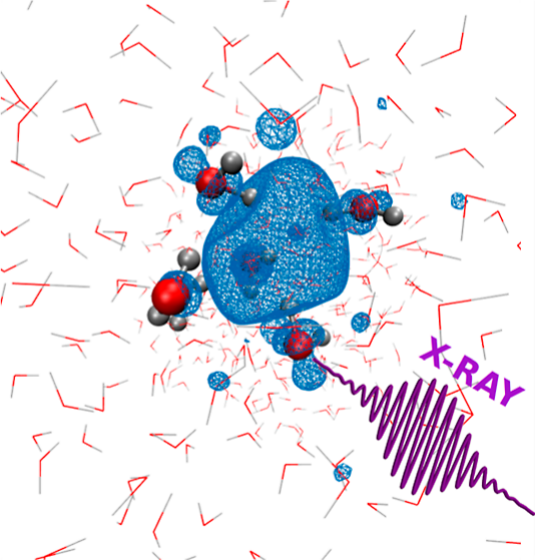

We present time-resolved X-ray absorption spectra of ionized liquid water and demonstrate that OH radicals, H_3_O^+^ ions, and solvated electrons all leave distinct X-ray-spectroscopic signatures. Particularly, this allows us to characterize the electron solvation process through a tool that focuses on the electronic response of oxygen atoms in the immediate vicinity of a solvated electron. Our experimental results, supported by ab initio calculations, confirm the formation of a cavity in which the solvated electron is trapped. We show that the solvation dynamics are governed by the magnitude of the random structural fluctuations present in water. As a consequence, the solvation time is highly sensitive to temperature and to the specific way the electron is injected into water.

## Introduction

The time scales of events involved in the radiolysis of water span from a few attoseconds, for the initial ionization event, to several femtoseconds and picoseconds for the formation of ions and radicals such as OH^–^, H_3_O^+^, and OH^•^.^1^ Water radiolysis also yields a substantial number of secondary electrons as byproducts, especially when water interacts with high-energy radiation (α-particles, β-, γ-, or X-rays).^2^ In liquid water, these electrons gradually lose energy and decelerate as they collide with the surrounding medium, spreading radiation-induced damage by exciting or ionizing other molecules. Some of these electrons may be captured by molecules within the medium, while others continue to decelerate until they have the ability to polarize the surrounding water molecules. In response to this polarization, water molecules orient themselves to create a cage around the electron, resulting in the formation of the solvated electron (e_aq_^–^).^1,3^ Several picoseconds after its formation, the solvated electron will recombine.^4−6^ The fundamental yet intriguing nature of this seemingly simple species—a single electron surrounded by water molecules—has sparked numerous studies since its discovery,^7^ which revealed important implications across a broad spectrum of fields, including radiation therapy,^3^ corrosion in nuclear reactors,^8^ and pollutant degradation.^9^

Due to challenges in its experimental characterization and theoretical modeling, the structure of e_aq_^–^ has been the subject of intense debate.^10^ The consensus places the electron at the center of a cavity created by 4–6 water molecules, each coordinating one of their hydrogens to the electron.^11^ Tracking the birth of this species is equally challenging owing to the short lifetime of e_aq_^–^’s precursor, which requires ultrafast techniques. Thus, many critical aspects of the electron solvation process remain unclear. Open questions include the solvation time scale, for which studies have reported values ranging from a few hundred femtoseconds to 2 ps,^5,6,12,13^ the influence of the preceding ionization mechanism,^6,14,15^ or the specific nature of e_aq_^–^’s immediate precursor,^5,12−19^ to name a few. At the origin of this general lack of consensus lies the inherent difficulty of disentangling the vibrational and electronic responses when using optical techniques.^20^ While e_aq_^–^ is rather transparent to X-rays, using computations of the K-edge X-ray absorption spectra (XAS), Li et al. recently showed that X-rays could be used to probe the presence of the cavity around e_aq_^–^.^21^ They suggested using time-resolved X-ray absorption spectroscopy (trXAS) to track changes in the solvation structure of e_aq_^–^ exploiting the high structural sensitivity of XAS.^22,23^ X-rays have already demonstrated their exceptional suitability for uncovering local transient structures in liquid water.^24,25^ But so far, experimental applications of X-ray spectroscopy to the solvated electron and its solvation shell remain absent. In this article, we report the most direct evidence of cavity formation with the help of trXAS. Thanks to a sophisticated all-electron methodology, we further deepen our understanding of how e_aq_^–^ and its environment affect one another during cavity formation.

## Methodology

### Experimental X-ray Absorption Measurements

**Figure 1:**
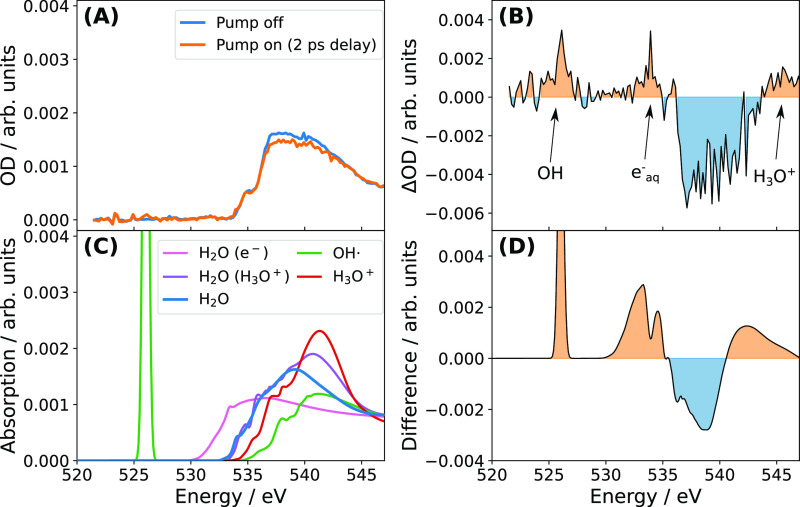
XAS of water radiolysis. (A) Experimental XAS for unpumped and pumped (after a 2 ps pump–probe delay) water. (B) Experimental differential XAS. (C) Theoretical XAS for solvated OH^•^ (green line), solvated H_3_O^+^ (red line), water molecules in the solvation shell of H_3_O^+^ (purple line), water molecules belonging to the cavity of e_aq_^–^ (pink line), and water molecules in the bulk (blue line). (D) Calculated difference XAS for water containing the aforementioned species.

**Figure 2:**
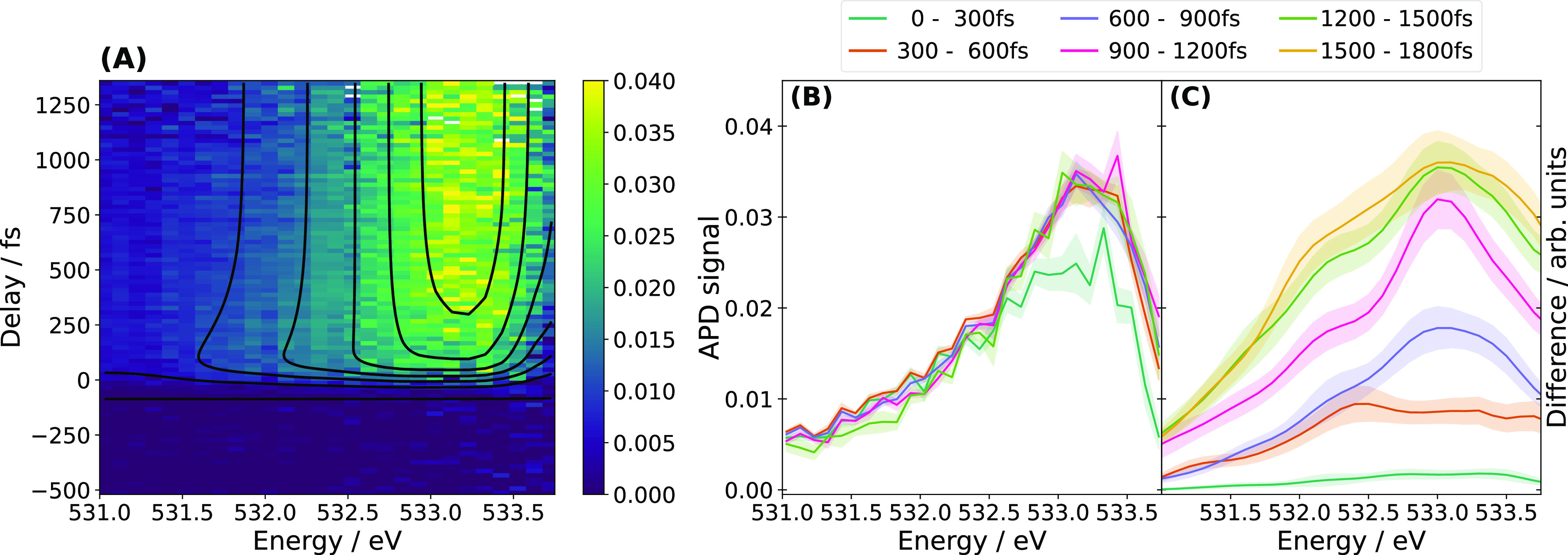
Transient X-ray spectroscopy of electron solvation in water. (A) Transient absorption signal from ionized liquid water after subtracting the static contribution from nonionized water (delay <0). (B) Same signal as in panel (A) but binned into time bins of 300 fs for comparison with theoretical trXAS difference spectra. (C) Calculated difference spectra at 300 K with a thermostat for different time bins after electron injection into the bulk water. The color-shaded areas indicate the standard error due to statistical sampling.

We recorded XAS for the different species involved in the strong-field ionization of liquid water employing an 800 nm pump and a tunable ∼30 fs ultrafast X-ray probe from the Linac Coherent Light Source X-ray free electron laser. The XAS shown in Figure 1 was measured for a fixed pump–probe delay in a thin water sheet jet (0.7 μm) in transmission mode, as described in detail in the Supporting Information. The thin sheet jet allowed us to measure an unpumped liquid water XAS that reproduced the synchrotron measurement reported in ref (26). The overall ionization fraction was estimated to be ∼1%. The trXAS shown in Figure 2 was obtained using total fluorescence yield as described previously.^27^

At the intensity of our pump pulse, the 800 nm strong-field ionization proceeds via a multiphoton process involving 8–9 photons, which is well above the vertical ionization potential of liquid water (11.33 eV)^28^—producing an electron with nonzero kinetic energy in the conduction band.

### Theoretical Methodology for Simulation of Electron Solvation

We model the electron solvation process using ring polymer molecular dynamics (RPMD) in combination with a neural-network force field trained with high-level ab initio electronic structure calculations. Without having to perform the unfeasible task of solving the time-dependent Schrödinger equation for the nuclear coordinates, the RPMD method incorporates some of the nuclear quantum effects that seem to be crucial for accurate modeling of the solvation mechanism.^29^ The employed neural-network force field was taken from an earlier work by Lan et al.^30^ It was parameterized for the ground state of an anionic box of 47 water molecules with periodic boundary conditions. The modeling thus represents the well-defined scenario of an excess electron injected into bulk water with zero additional energy. The training data set for the neural network included forces and energies calculated at the MP2 level of theory. This level of electronic structure is considered to be state-of-the-art for the study of liquid water^31^ and the formation dynamics of the solvated electron.^32^ To identify cavity formation, we calculated the spin density at the density functional theory level of theory at selected snapshots of the simulation (every 100 fs). This was necessary, as the RPMD calculations only included the solvated electron implicitly through the neural-network force field.

### Theoretical Methodology for the Calculation of XAS

To model the X-ray signatures for all relevant species in the ionization of liquid water, we sampled structures from simulations of the solvated species. For e_aq_^–^ and H_2_O, we took structures from the RPMD simulations. Because no neutral-network-based force field was available for OH^•^ and H_3_O^+^, we employed ab initio MD simulations for these species (see Supporting Information for simulation details and the procedure that was used to sample geometrical structures from these simulations). We calculated the XAS of these species with our in-house software package XMOLECULE.^33,34^ To adequately describe the spectral region around the X-ray absorption edge that involves very diffuse orbitals, we employed a Gaussian basis set that was extended with additional Kaufmann basis functions.^35^ From the discretized pseudocontinuum that results from this Gaussian basis set, we computed the absorption spectra for the electronic continuum via the Stieltjes-imaging procedure.^36^ A more detailed description of the calculation of the XAS can be found in Supporting Information.

## Results and Discussion

### Experimental and Theoretical XAS

Previously, some coauthors of this paper noted a feature in the pre-edge region of ionized water (531.0–533.7 eV) when tracking the formation and reaction of H_2_O^+^ using X-rays.^27^ This feature corresponds to none of the species participating in the reaction H_2_O^+^ + H_2_O → OH^•^ + H_3_O^+^ and its time scale aligns with previous studies on electron solvation, so the authors tentatively assigned it to the formation of e_aq_^–^. In Figure 1, we show new experimental and theoretical results on water radiolysis, confirming that e_aq_^–^ indeed produces a distinct feature in the pre-edge region of the XAS. In panel B, we present experimental data for the differential static XAS between bare water (pump off) and ionized water 2 ps after interaction with the strong-field pump laser. The difference spectrum exhibits an increased absorption peak at 526 eV, a broad absorption gain in the 530–534 eV range, a depletion of the absorption between 535–541 eV, and a slight absorption increase around 544 eV. This differential signal reflects the contributions of the various species involved in the radiolysis of liquid water. At this pump–probe delay, H_2_O^+^ has already decayed via proton transfer to a neighboring water molecule,^27^ and the recombination of free electrons with holes has not yet occurred. This leaves OH^•^ radicals, H_3_O^+^ cations, and e_aq_^–^ as the main contributors to the XAS difference spectra.

In order to assign the key features of the XAS difference spectra from Figure 1B to the aforementioned radiolysis byproducts, we calculated the XAS values for each of these species individually. In Figure 1C, we show the calculated XAS of solvated OH^•^ (green line), solvated H_3_O^+^ (red line), water molecules in the solvation shell of H_3_O^+^ (purple line), water molecules belonging to the cavity of e_aq_^–^ (pink line), and water molecules in the bulk (blue line). For OH^•^, we can observe a strong resonance feature at 526 eV^27^ associated with a transition between the core level and the singly occupied valence orbital of the radical. In the radical, the remaining absorption edge is pushed to higher excitation energies compared to the absorption edge of bare water. A similar shift of the main absorption edge to higher energies also appears for H_3_O^+^, followed by a strong increase in absorption in the postedge region (∼541 eV).^37^ The presence of H_3_O^+^ may also influence water molecules in its solvation shell since it temporarily creates superstrong hydrogen bonds with neighboring water molecules.^38^ We therefore also inspect the X-ray absorption of water molecules in the vicinity of H_3_O^+^. As can be seen, at lower absorption energies (pre- and main-edge), these water molecules, H_2_O(H_3_O^+^) shown in purple, have an absorption spectrum very similar to that of bulk water. At somewhat higher absorption energies, an additional feature at about 541 eV similar to the one in H_3_O^+^ appears. The resulting shifts can be interpreted in terms of the different orbital hybridizations in H_3_O^+^ and its neighboring water molecules.^39^ For water molecules belonging to the cavity of e_aq_^–^, we observe a strong absorption gain in the pre-edge region of the XAS (∼533 eV). Li et al.^21^ recently showed that the solvated electron penetrates the antibonding orbitals of water molecules from its cavity in a similar way as in the formation of hydrogen bonds, thus producing the observed absorption gain in the pre-edge region of the XAS.^21^ These last two species in particular—water molecules from the cavity of e_aq_^–^ and water molecules in the solvation shell of H_3_O^+^—highlight the remarkable sensitivity of XAS to the local hydrogen-bond environment of water molecules.^25,40^

Each of the discussed species induces a change in the X-ray absorption spectrum of liquid water that can be modeled with
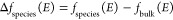
1Here, Δ*f* (*E*) represents the XAS difference induced by a given species. The sum of overall considered species
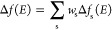
2reveals a superposition model for the XAS difference of ionized liquid water, where *w*_s_ represents an appropriate weight for the relative amount of the species s. For example, we observe that on average, about 4.5 water molecules are part of the cavity surrounding the solvated electron, whereas for each solvated electron, only one OH^•^ should be considered. This model implies that all water molecules, excluding those belonging to the solvated electron’s cavity or the solvation shell around H_3_O^+^, exhibit largely unchanged XAS and that ionization is diluted enough to treat these species separately. We refer here to ref (21) for a more detailed explanation of the negligible contribution to the XAS of water molecules outside the cavity of e_aq_^–^.

Figure 1D illustrates the modeled XAS difference spectra arising from the superposition of calculated XAS-difference spectra for all the species presented in Figure 1C. The assigned relative weights are *w*_s_ = 1 for H_3_O^+^ and OH, *w*_s_ = 3 for water molecules in the solvation shell around H_3_O^+^, and *w*_s_ = 4.5 for water molecules in the cavity of e_aq_^–^. As can be seen, this model successfully reproduces all of the key features of the experimental difference spectra. The good agreement between theory and experiment allows us to assign the features with an absorption gain in the pre-edge region to individual species. Specifically, we can assign the peak at 526 eV to the strong resonance of OH^•^^27^ and the absorption gain in the range 530–534 eV to the cavity around e_aq_^–^. The latter confirms that e_aq_^–^ cavities produce a distinct signal in the pre-edge region of the XAS—as previously proposed by Li et al.^21^—thereby demonstrating the potential of X-rays to trace cavity formation.

### Electron Solvation: Experimental and Theoretical Results

In the following, we focus on the time evolution of the XAS in the region 530–535 eV, as it captures the formation of cavities around e_aq_^–^ in liquid water. Specifically, the rise in absorption intensity in this range reflects the formation of cavities. We show both experimental and theoretical results for the trXAS of e_aq_^–^ in Figure 2. The transient signal that appears in Figure 2A, characterized by a primary peak at 533.3 eV, is a direct consequence of cavity formation in liquid water. We obtained a cavity formation time scale of 0.26 ± 0.03 ps through a global fit of the experimental signal with a universal time constant.^27^

To calculate the trXAS difference spectra from the simulations, we inspected the resulting cavities as a function of time after the electron injection. We grouped all snapshots with fully formed cavities into time bins and averaged the calculated XAS over the structures in each time bin.

The resulting difference spectra in Figure 2C show how electron solvation increases X-ray absorption around 533 eV on a time scale of about 1300 fs. In Figure 2B, we compare these results with the experimental difference spectra employing the same time-binning. While the change in the XAS is qualitatively similar in both experiment and theory, we observe a significant discrepancy in the time scale at which this spectral change appears. The smaller time scale for the spectral change in the experiment directly reflects the fact that cavity formation seems to proceed considerably faster in the experiment than in the simulations.

The discrepancy might stem from an unaccounted local temperature increase during the injection of the excess electron into liquid water in the experiment. Due to the substantial amount of energy deposited by the strong-field pump, the ionized electron has enough initial kinetic energy to locally increase the temperature through inelastic collisions, which would lead to faster solvation.^41^ If we assume as a lower estimate an initial electron kinetic energy of 1 eV, 1% ionization probability, and full equilibration, the resulting temperature rise amounts to ∼13 K. However, our simulations model the situation of an excess electron injected into bulk water with zero kinetic energy. As we will show, this difference can explain the discrepancy in the time scale of the solvation process. Another possible origin for the slower solvation in the simulation could be an artificial dampening of the dynamics caused by the thermostat.^42^ Using a thermostat in a nonequilibrium process, while counterintuitive, becomes necessary due to the limited capacity of our small simulation box to transport heat between the solvation region and the bulk. The thermostat prevents such an unphysical accumulation of heat that could alter the dynamics. To test the effects of both temperature and the thermostat on the solvation dynamics, we performed two additional sets of RPMD simulations: one with a higher temperature and another without a thermostat.

### Electron Solvation Mechanism

**Figure 3:**
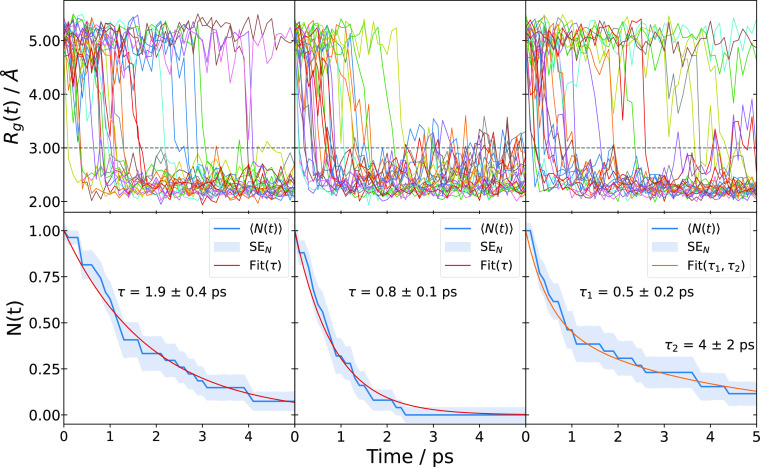
Solvation time scales from simulated trajectories at 300 K with a thermostat (left column), at 340 K with a thermostat (central column), and at 300 K without a thermostat (right column). The top row shows the evolution of the gyration radius for the different trajectories. The dashed line at 3 Å marks the threshold below which we assume a cavity to be formed. The bottom row shows the evolution of the ratio of trajectories in which cavity formation has not (yet) taken place. The orange curve shows a monoexponential (left and central columns) or biexponential fit to the data.

After the insertion of the excess electron in the simulation, the electron is almost completely delocalized over the entire simulation box. After a certain simulation time, the spin density contracts and localizes, which is accompanied by the formation of cavity structures. Figure 3 shows the gyration radius of e_aq_^–^ (top panel) and the fraction of cavities that have not formed (bottom panel) as a function of time for the three different situations considered: *T* = 300 K with a thermostat, *T* = 340 K with a thermostat, and *T* = 300 K (initial temperature) without a thermostat. We observe for the two latter scenarios considerably faster solvation dynamics, especially at early times. In contrast to the thermostated simulations, where cavity formation follows a monoexponential trend, the simulation results for *T* = 300 K without a thermostat are considerably better fitted with a double exponential (see Supporting Information). Aside from this long-time behavior of the nonthermostated calculation, both new sets of calculations reveal subpicosecond solvation time scales (τ = 0.8 ± 0.1 ps for *T* = 340 K with a thermostat and τ_1_ = 0.5 ± 0.2 ps for *T* = 300 K without a thermostat) in contrast to the original calculation at *T* = 300 K with a thermostat (τ = 1.9 ± 0.4 ps). The observed acceleration is compatible with the so-called “trap-seeking” mechanism,^43−46^ in which random orientational fluctuations govern the solvation time scale. In this mechanism, the electron remains in a delocalized state, waiting for a favorable configuration of water molecules—a trap—to randomly occur. The electron then collapses into this trap, forming a cavity around it. Enhancing orientational fluctuations by increasing the temperature or by deactivating the thermostat thus results in more traps and faster solvation. One proposed candidate for such a trap is a broken hydrogen bond,^32,47,48^ whose concentration is dependent on random fluctuations and temperature.^25,40^

**Figure 4:**
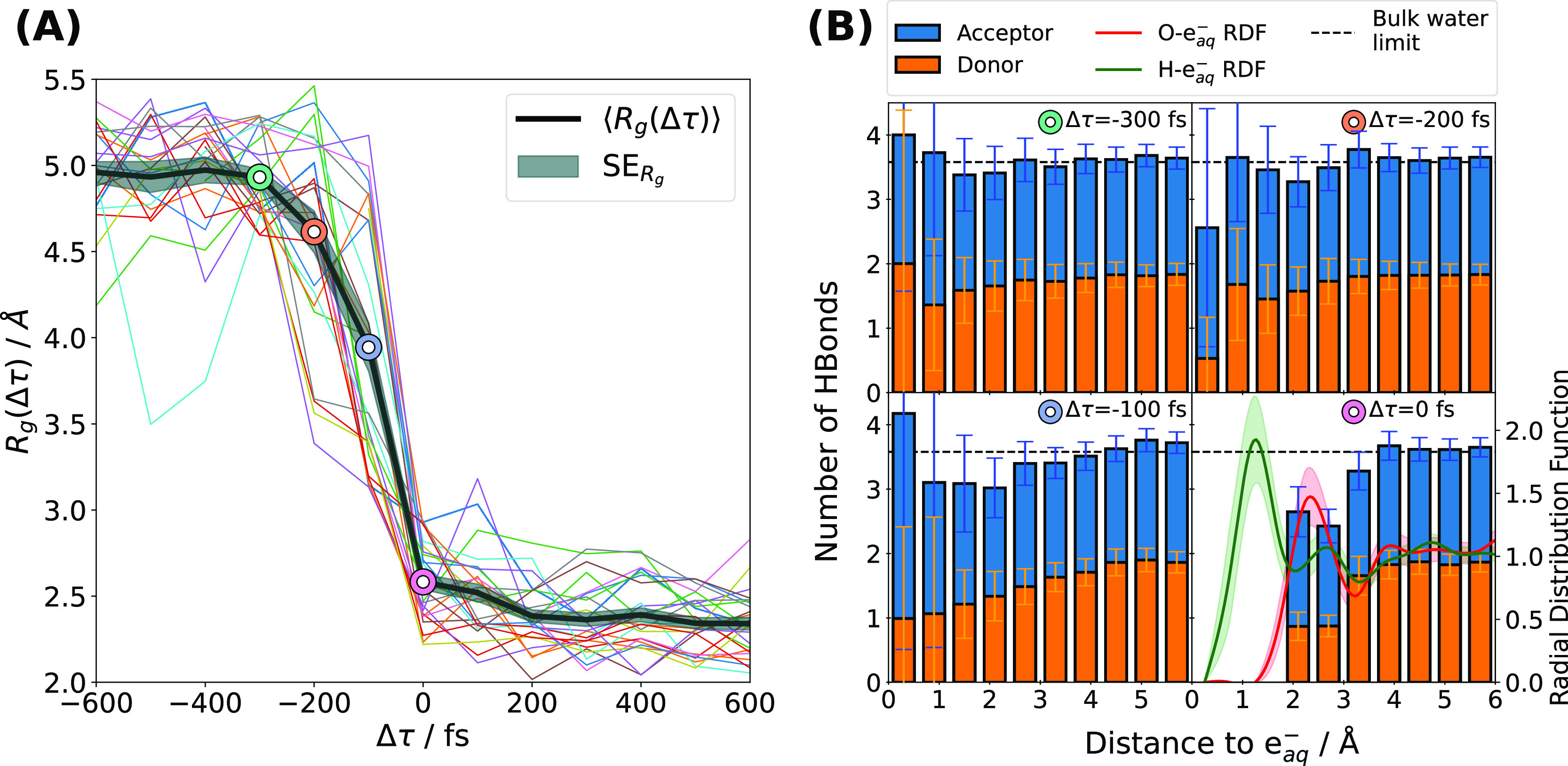
Evolution of the solvation process as a function of the relative formation time Δτ = *t* – τ, where τ represents the cavity-formation time of each trajectory. (A) Gyration radius of the electron spin density, *R*_g_(Δτ), for the various sampled trajectories. (B) Number of hydrogen bonds per water molecule (donors in orange, acceptors in blue) as a function of the distance from the spin center of mass of the electron for different Δτ (see colored circles). The dashed horizontal line at 3.58 marks the total average number of hydrogen bonds per water molecule in bare water at room temperature.^51^ For Δτ = 0 fs (bottom-right panel), we also include the radial distribution function for oxygen and hydrogen (red and green curves, respectively).

In Figure 3, different trajectories show different onset times for electron localization. However, once the collapse of the gyration radius starts, all trajectories follow a similar trend. To make this trend more apparent, we have plotted in Figure 4A the gyration radius of each trajectory against its relative formation time Δτ = *t* – τ, where τ is the 100 fs time step where the gyration radius *R*_g_(*t*) of each trajectory shrinks below 3 Å. Following the random onset of solvation, governed by the trap-seeking mechanism, we observe a concordant collapse of the gyration radius of the spin density across all trajectories for the three cases studied (see Supporting Information for the trajectories at 340 K and the trajectories without a thermostat). This indicates that cavity formation has entered a digging stage, where orientational fluctuations are no longer the main driving force. To further investigate the nature of this hybrid trap-seeking/cavity-digging^49^ solvation mechanism, we analyzed the hydrogen-bond network of the water molecules surrounding e_aq_^–^, since its disruption is linked to the solvation process.^47^Figure 4B shows the number of hydrogen bonds per water molecule as a function of the distance from where the spin center of mass of e_aq_^–^ will be when the cavity forms (Δτ = 0 fs). The data reveal how the disruption of the hydrogen-bond network is tied to the collapse of the gyration radius. When the electron is still delocalized at Δτ = −300 fs, the hydrogen-bond network around the electron resembles that of bulk liquid water. At Δτ = −100 fs, one can see how the localization of the electron goes together with a local breakdown of the hydrogen-bond structure, affecting mainly the number of donor hydrogen bonds in close vicinity of the future center of the spin density. This disruption propagates outward as the electron shrinks and culminates in the formation of a solvation layer in the region of 2–3 Å around the electron at Δτ = 0 fs. Water molecules in the solvation layer can only donate one of their hydrogens to other water molecules; the other hydrogen points toward the center of the cavity and coordinates with the electron, as can be seen in the histogram and the radial distribution functions of oxygen and hydrogen atoms around the spin center at Δτ = 0 fs (lower-right panel of Figure 4B). Random fluctuations alone cannot explain such a large and coordinated disruption of the hydrogen-bond network, which supports the idea that the electron is actively digging its own cavity.^50^

## Conclusions

Our study shows that trXAS is a sensitive probe of the cavity of e_aq_^–^ and can be used to track electron solvation. It is important to stress that X-ray absorption does not probe the solvated electron directly but rather probes the structural changes in water induced by the solvated electron and is thereby complementary to earlier works addressing the question of electron solvation using other methods. The fact that e_aq_^–^ leaves a noticeable change in the X-ray absorption is therefore incompatible with the concept of a delocalized solvated electron as was hypothesized earlier.^52^ Our results are evidence for cavity formation induced by e_aq_^–^. According to our trXAS measurements, the cavity around e_aq_^–^ forms in 0.26 ps after the injection of an excess electron in liquid water at room temperature. The simulations at 300 K with a thermostat, however, exhibit a much longer solvation time scale of 1.3 ps. Our analysis suggests that random orientational fluctuations play a major role in determining the solvation time scale by affecting the appearance of suitable traps for electron localization. The resulting picture of electron solvation in water is that of an electron that waits for a suitable trap to randomly appear and then starts to actively dig its cavity, causing disruption of the surrounding hydrogen-bond network. While the trap-seeking mechanism is influenced by structural fluctuations, which are notably influenced by temperature and the electron’s specific preparation in water, the subsequent collapse into a cavity occurs relatively fast (<100 fs). The elucidation of X-ray spectroscopic signatures for e_aq_^–^, OH^•^, and H_3_O^+^ suggests that future studies will be able to provide more insight into ionization dynamics, proton transfer, and proton hydration in water. In particular, further insight may be gained by studying electron solvation as a function of sample temperature and ionization mechanism.

## Supporting Information Available

Experimental setup at ChemRIXS; theoretical methodology; and additional simulation results (PDF)
