# Supplementary material for: Tracking Cavity Formation in Electron Solvation: Insights from X-ray Spectroscopy and Theory
Source: J Am Chem Soc. 2024 Jan 25;146(5):3262–9. doi: 10.1021/jacs.3c11857 (PMC10859959; doi:10.1021/jacs.3c11857)
Supplement: Supplementary file 1 — ja3c11857_si_001.pdf [file ja3c11857_si_001.pdf]

Supporting Information: Tracking Cavity Formation in Electron Solvation: Insights from X-ray Spectroscopy and Theory

Arturo Sopena Moros,[†,‡‡] Shuai Li,[‡,‡‡] Kai Li,[‡,¶] Gilles Doumy,[‡] Stephen H Southworth,[‡] Christopher Otolski,[‡] Richard D. Schaller,[§,∥] Yoshiaki Kumagai,[⊥] Jan-Erik Rubensson,[#] Marc Simon,[@] Georgi Dakovski,[△] Kristjan Kunnus,[△] Joseph S Robinson,[△] Christina Y Hampton,[△] David J Hoffman,[△] Jake Koralek,[△] Zhi-Heng Loh,[∇] Robin Santra,[†,††] Ludger Inhester,[*,†] and Linda Young[*,‡,¶]

†*Center for Free-Electron Laser Science CFEL, Deutsches Elektronen-Synchrotron DESY, Notkestr. 85, 22607 Hamburg, Germany*

‡*Chemical Sciences and Engineering Division, Argonne National Laboratory, Lemont, Illinois 60439, United States*

¶*Department of Physics and James Franck Institute, The University of Chicago, Chicago, Illinois 60637, United States*

§*Center for Nanoscale Materials, Argonne National Laboratory, Lemont, Illinous 60439, United States*

∥*Department of Chemistry, Northwestern University, 2145 N. Sheridan Rd., Evanston, IL 60208*

⊥*Department of Applied Physics, Tokyo University of Agriculture and Technology, 184-8588 Tokyo, Japan*

#*Department of Physics and Astronomy, Uppsala University, Box 516, SE-75120 Uppsala, Sweden*

@*Sorbonne Université, CNRS, Laboratoire de Chimie Physique-Matière et Rayonnement, LCPMR, F-75005, Paris, France*

△*LCLS, SLAC, Menlo Park, California 94025, United States*

∇*School of Chemistry, Chemical Engineering and Biotechnology, and School of Physical and Mathematical Sciences, Nanyang Technological University, Singapore 637371, Singapore*

††*Department of Physics, Universität Hamburg, Notkestr. 9, 22607 Hamburg, Germany*

‡‡*A.S.M and S.L. contributed equally to this work*

E-mail: ludger.inhester@cfel.de; young@anl.gov

Contents

S-I Experimental Setup at ChemRIXS **S-1**

S-I.1 Incoming energy calibration and normalization S-3

S-I.2 XAS of liquid water S-4

S-I.3 Transient XAS of ionized water at 2 ps delay S-4

S-I.4 Liquid Jet S-5

S-II Theoretical Methodology **S-7**

S-II.1 Dynamics and characterization of electron solvation S-7

S-II.1.1 Detection of Cavities S-8

S-II.1.2 Characteristic Formation Times: Fitting Procedures S-10

S-II.1.3 Hydrogen-Bond Network Analysis S-13

S-II.2 Molecular Dynamics Simulations for H_2O, OH $\cdot$, and H_3O^+ S-13

S-II.3 Calculation of X-ray Absorption Spectra S-14

S-II.3.1 Sampling of structures for XAS calculations S-17

S-III Additional Results **S-19**

References **S-19**

S-I Experimental Setup at ChemRIXS

The optical ionization pump-soft x-ray probe measurements were performed at the ChemRIXS endstation of the Linac Coherent Light Source (LCLS) x-ray free-electron laser (XFEL). Fig. S1 shows a schematic of the experimental setup. The 800 nm output of a Ti:sapphire amplifier was focused onto a 700 nm-thick converging liquid sheet jet target[1,2] to ionize liquid water by strong-field ionization. The surface of the liquid jet was oriented normal to the incident 800 nm ionization pump and soft x-ray probe beams, with a crossing angle of ∼1°

between the two beams. The pump laser was combined with the x-rays by a holey mirror upstream of the target and skimmed out by another holey mirror downstream of the target. With a pulse duration of 55 fs, a focal spot diameter (FWHM) of 75 μm and a pulse energy of 0.5 mJ, the peak intensity of the pump laser was 1.3×10^{14} W/cm^2 at the interaction region. The intensity and polarization of the 800 nm pump laser could be controlled by an ND filter and a $\lambda/2$ waveplate in the 800 nm delivery path. The 20 fs XFEL pulse becomes stretched to ~ 30 fs after being monochromatized to a spectral width of 0.1 eV. It was then focused by a pair of Kirkpatrick-Baez (KB) mirrors to a spot size (FWHM) of 32(H) $\times$ 130(V) μm^2 on the target. The focal point was located between the arrival time monitor (ATM)[3] and the target interaction point (IP), to provide sufficient x-ray intensity on the ATM screen. The ATM, as shown in Fig. S2, positioned 1.5 m upstream of the target, cut off about half of the x-ray beam before the focus and recorded the relative arrival times of the optical laser and the XFEL pulses. For the experimental results shown in Fig. 1 in the main manuscript, the optical-XFEL timing jitter limited the effective time resolution to $\sim$65 fs. The time delay between the optical pump and x-ray probe pulses at the interaction region was controlled by the upstream global delay on the optical arm. The local optical delay stage upstream of the ATM (shown in Fig. S1) is employed to fulfill the operational requirements of the ATM.

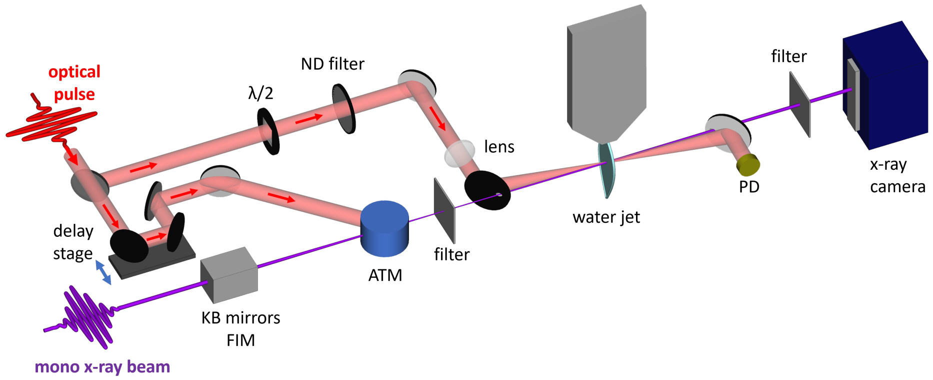

Figure S1: Schematic layout of the experimental setup at the ChemRIXS beamline: 800 nm strong-field ionization pump, time-delayed x-ray probe, arrival time monitor and transmission x-ray camera.

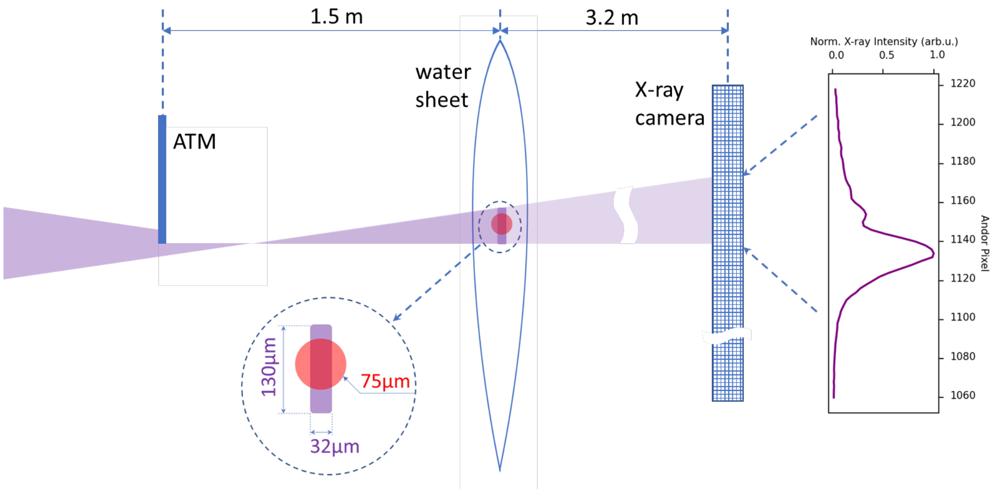

Figure S2: Optical layout for arrival time monitor (ATM) and XAS measurement. The x-ray was partially cut off by the ATM screen and focused upstream of the water jet; the transmitted x rays were recorded by the x-ray camera. The x-ray intensity distribution from the x-ray camera is shown on the right. The relative sizes of the x-ray (purple) and laser spots (red) on the water jet is shown in the dashed circle.

S-I.1 Incoming energy calibration and normalization

The incident energy (I_0) of the monochromatic x-ray pulse was recorded by fluorescence intensity monitors (FIM) installed above the KB mirrors.[4] The transmitted energy (I) of x-ray pulse was recorded by a camera (Andor Newton SO DO940P) in full-vertical-binning mode, where the 2D image was projected to a vertical 1D array. The 1D array maps the transmitted x-ray intensity from various points of the jet leaf from upstream to downstream in the interaction region, which makes it possible to extract the x-ray absorption spectrum (XAS) for various thicknesses and various pump laser intensities. Based on the Beer-Lambert law $\Delta \mathrm{A} = \log(I_0/I)$, the XAS of the water sheet, shown in Fig. S4, can be easily obtained by subtracting the no-sample XAS from the with-sample XAS. The correlation between I_0 and I without sample is shown in Fig. S3. The XAS is recorded by scanning the x-ray photon energy with a step size of 0.2 eV, with more than 1000 shots per step. The x-ray photon energy scan is performed by scanning the undulator gap in coordination with the monochromator; this gave about 50% variation of the averaged x-ray pulse power over a

relatively large photon-energy range, 520 to 550 eV. The energy of the monochromator readout was calibrated by the pre-edge feature of liquid water absorption.

S-I.2 XAS of liquid water

The water XAS shown in Fig. S3 was taken by a synchronized undulator-monochromator scan near the oxygen K-edge region. The background absorption was taken without the water jet. Fig. S4 shows the experimental results here reproduce the reference water XAS spectrum obtained by Meibohm *et al.*[5]

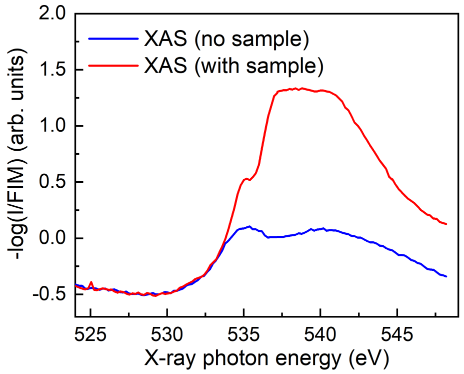

Figure S3: The XAS with and without water sample. The arbitrary vertical scale is offset from zero in the water window because different types of detectors were used to measure the incident and transmitted x-ray intensity.

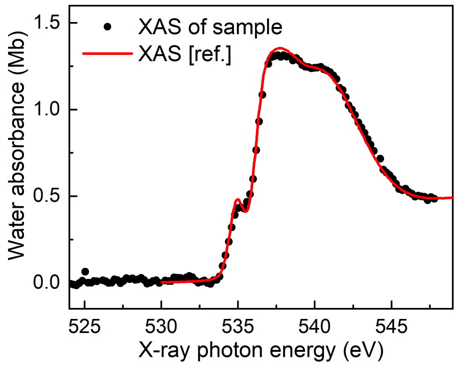

Figure S4: The XAS of water sample. Black dot is experimental data, red curve is the XAS spectrum at 23°C.[5]

S-I.3 Transient XAS of ionized water at 2 ps delay

The transient x-ray absorption spectrum ΔA of strong-field ionized water was recorded with a pump-probe delay at 2 ps. A trigger kicks out the pump laser 30 ns after the x-ray probe pulse and was used to turn the pump laser 'off'. The ratio of the number of shots with pump-on and with pump-off was 1:1. Fig. S5 shows the x-ray/optical laser overlap - together with the averaged x-ray transmission signal as a function of X-ray camera pixel number. The

center position of the laser projected to the camera is determined by the measured ΔA signal. The relative widths of the x-ray and laser were determined by knife-edge scans. By selecting the pixel number of the transmitted x-ray signal on the camera, we select the ΔA for specific pump laser intensities. The purple region corresponds to a pump laser intensity of $\sim 10^{13}$ W/cm², which is $\sim 7\%$ of the peak pump intensity and similar to that used in earlier study of strong-field-ionized liquid water.[6] This yields an ionization fraction of $\sim 1\%$. We note that the intensity required for a given ionization fraction is inversely proportional to the target thickness.

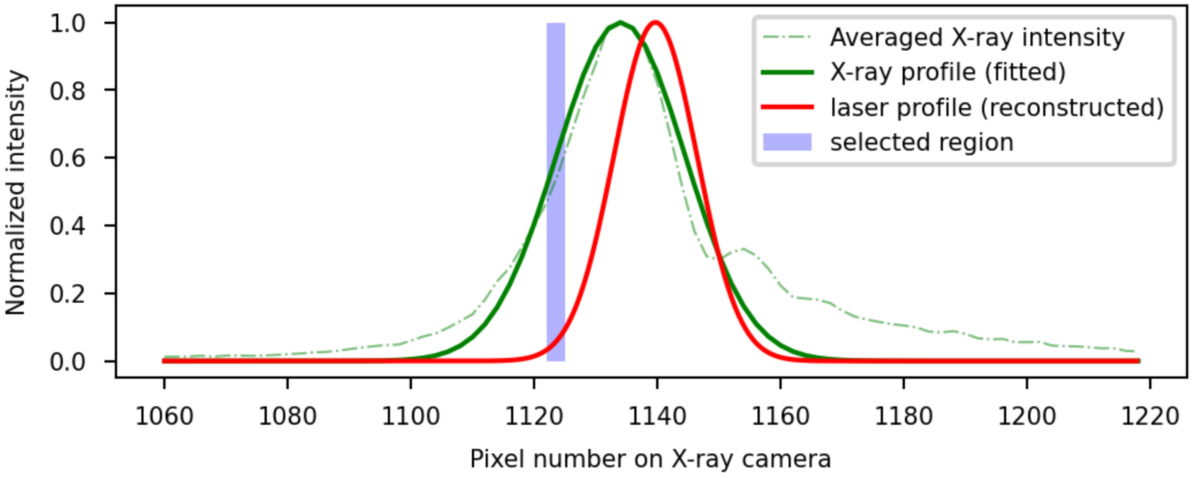

Figure S5: The spatial overlap between X-ray and laser. Green dash-dot line shows the averaged X-ray transmission intensity on the X-ray camera, from which the X-ray profile was fitted as shown by the green solid line. The integral of the absolute ΔA over $525 - 540$ eV region on each X-ray camera pixel was used to fit the center position of the pump laser. The spot sizes were measured by knife-edge scans and the Gaussian laser profile was reconstructed as shown by the red solid line. The transient absorption spectrum at 2 ps shown in Fig. 1B in the main manuscript is from the purple region.

S-I.4 Liquid Jet

The thin water jet avoids saturation of absorption by the target. The averaged thickness over x-ray spot is estimated to be ~ 700 nm as deduced by comparison with the absolute absorbance of Ref. 7 at the water main edge. As shown in Fig. S6(a), the thickness of the

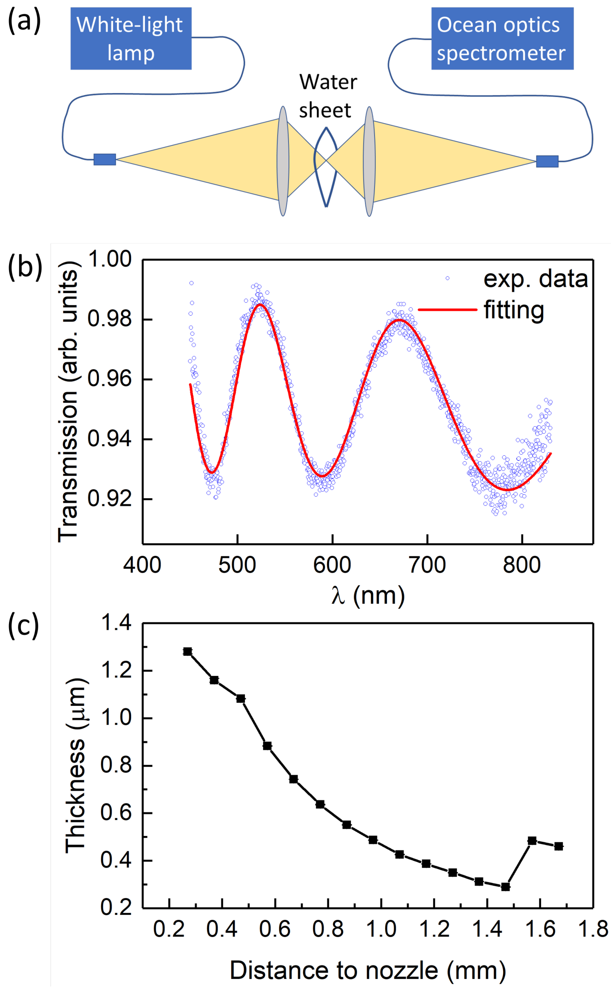

Figure S6: Measurement of the water sheet jet thickness. (a) Schematic for the white-light interferometry, (b) Typical transmission recorded by the spectrometer, (c) Water sheet thickness fitted by the equation 1, as a function of the distance to the nozzle.

water jet is also measured through the transmission thin-film interferometry[8] at Argonne National Laboratory. The white light from a white light lamp (Thorlabs, OSL2 Fiber Illuminator) is focused on the water sheet and the transmitted spectrum is measured with a spectrometer (Ocean optics, USB2000). Here the flow rate of the HPLC pump was set to 1.75 ml/min, the pressure was 275 psi and the thickness of water sheet as a function of the distance from the nozzle is shown in Fig. S6(c). Due to the node at the bottom of the first water sheet leaf, the thickness there shows a rapid increase. Fig. S6(b) shows an example of fitting the water-sheet thickness from the transmission of the white light spectrum, in the

following fitting function[8] t and r are the transmission and reflection of water, respectively, n is the refractive index of water, d is the thickness of water sheet. The parameters of a, c, k are added to get better fitting which are not sensitive to the oscillation period and therefore do not affect the measured thickness.

$$T = c + k\lambda + \frac{t^4}{1 + r^4 + 2ar^2 \cos \frac{4\pi nd}{\lambda}} \tag{1}$$

S-II Theoretical Methodology

S-II.1 Dynamics and characterization of electron solvation

The modelling of the solvation dynamics is based on a force field implemented via a neural network (through the n2p2 package[9]). This neural-network force field was trained at the restricted/unrestricted MP2 level of the electronic ground state of neutral/anionic water with a triple-zeta quality correlation-consistent basis set as implemented in CP2K.[10] For more information on the training of the machine learning potential (MLP), see the SI from Ref. 11. The RPMD simulations were performed using 47 water molecules, with 32 beads for each atom, in a box with an edge length of 11.3 Å and periodic boundary conditions. The propagation was performed using the i-PI code[12] with a 0.25 fs time step. With a similar approach as the one used by Lan *et al.*, we begin by launching a simulation of neutral water for 5 ps at a given temperature (300 K or 340 K). Once these calculations with neutral water finished, the water box was considered to be equilibrated. The last snapshot of each of these trajectories was used as the starting point for a trajectory of the electron solvation dynamics. To introduce the excess electron in the simulation, we simply had to switch to the neural-network force field trained for the anionic water box and run the calculation for another 5 ps. We launched three sets of trajectories of the electron solvation dynamics, one at 300 K with a thermostat, one at 340 K with a thermostat, and another at 300 K with no thermostat. For the thermostated calculations, we employed an SVR thermostat (all parameters for

the thermostat can be found in the aforementioned Ref. 11). We launched between 34 and 37 trajectories for the three different sets of calculations. During the dynamics, some of these trajectories failed because they explored configurations for which the force field was not properly trained, so we had to discard them. In the end, we obtained 27 viable trajectories for the calculations at 300 K with a thermostat, 25 for the calculations at 340 K with a thermostat, and 26 for the calculations at 300 K without a thermostat. All files necessary to replicate these calculations, such as the i-PI input files and the files containing the parametrization of the neural-network force field, can be also found in the SI of Ref. 11.

S-II.1.1 Detection of Cavities

To identify the formation of cavities, we followed the same analysis procedure as Lan *et al.*[11] That is, from selected snapshots of the RPMD trajectories, we calculated the spin density using density functional theory with the CP2K code.[10] We employed the PBE(α) exchange-correlation functional with 40% Hartree-Fock exchange, which has been shown to provide a satisfactory description of the solvated electron, particularly in terms of producing a spin density comparable to that obtained using MP2, see SM from Ref. 11. Because these calculations are very demanding, we calculated the spin density only for snapshots of the centroid at every 100 fs. We observe that the spin density (and thereby the density of the excess electron) is initially completely delocalized throughout our simulation box. Only at some later time, the spin density localizes somewhere, which is accompanied by the formation of a cavity around the solvated electron. An example of such a formed cavity can be seen in Fig. S7.

Once we obtained the spin density from the DFT calculation, we shifted the periodic boundary conditions such that the spin density is minimal on the surfaces of the simulation box. We then computed the center of the spin density $\rho^s(\mathbf{r})$,

$$\mathbf{r}_c = \frac{\int \rho^s(\mathbf{r})\mathbf{r}dr^3}{\int \rho^s(\mathbf{r})dr^3}, \tag{2}$$

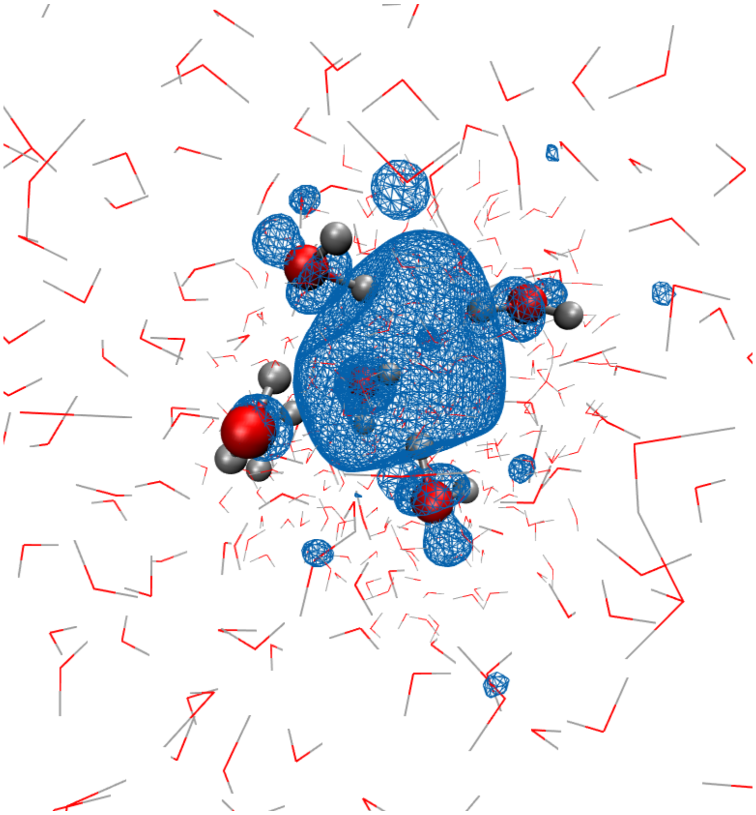

Figure S7: Spin density of the solvated electron for an isovalue of 0.0008. Water molecules represented with spheres belong to the first solvation layer. As can be seen, spin density can be found not only in the central excluded volume (the cavity), but also on the oxygen atoms of the first and even second solvation layer.

and the gyration radius by diagonalizing the gyration tensor

$$S_{mn} = \frac{\int (\mathbf{r} - \mathbf{r}_c)_m (\mathbf{r} - \mathbf{r}_c)_n \rho^s(\mathbf{r}) dr^3}{\int \rho^s(\mathbf{r}) dr^3}, \tag{3}$$

where m and n refer to the different x, y, z Cartesian components. We consider that a cavity has formed when the gyration radius is less than 3 Å. From this point, we can identify all water molecules that belong to the cavity of the solvated electron. To do so, we construct the isosurface of the spin density for an isovalue of $\rho_s = 0.001$. Generally, as soon as the electron has localized, such an isosurface encloses one large volume and several smaller volumes, i.e., spin bubbles. We focus on the isosurface associated with the largest volume and identify water molecules within a certain distance from this isosurface as belonging to the cavity.

Figure S7 illustrates a typical example, where one can see five water molecules that are within 1.4 Å from the isosurface of the largest spin bubble. For illustration purposes the depicted isosurface has a somewhat smaller isovalue of 0.0008.

The results for the gyration radius from all trajectories as a function of time are shown in Fig. S8. We have included the results of a global fit to the three sets of calculations to an exponential law where the baseline, $R_g(\infty)$, represents the asymptotic gyration radius of each set of trajectories. For the calculation at 300 K and without thermostat, a simple monoexponential fit yielded considerably worse results at early times compared to the fits for the thermostated calculations (see bottom panel of Fig. S8). However, using a biexponential fit produced better results. Both fits are included in the lower panel of Fig. S8

S-II.1.2 Characteristic Formation Times: Fitting Procedures

Given that the initial delocalization of the electron in our simulations depends on the box size, potentially influencing the solvation timescale obtained through gyration radius fitting, we performed an additional analysis for a more in-depth analysis of the characteristic times of the solvation process. For each trajectory, we adopted a binary criterion and assigned a value for each of the two possible states of the cavity at any given time, formed or not formed. A cavity is considered to be formed if the gyration radius at a given time is less than 3 Å. We assign a value of 1 to trajectories without a cavity ($R_g > 3$ Å), and a value of 0 to those with a cavity ($R_g < 3$ Å). The average of all trajectories corresponds to the fraction of trajectories in which, at a given time, cavity formation has not yet taken place. The results, together with a global fit to an exponential function, are shown in Fig. S9. As in the previous case, for the nonthermostated calculation, we obtained a better fit using a biexponential function rather than a monoexponential function. In this case, the improvement of the fitting is even more apparent. All global fits shown in Figure S8 and Figure S9 were performed using the lmfit library[13] together with bootstrapping to estimate standard error of the fit parameters.

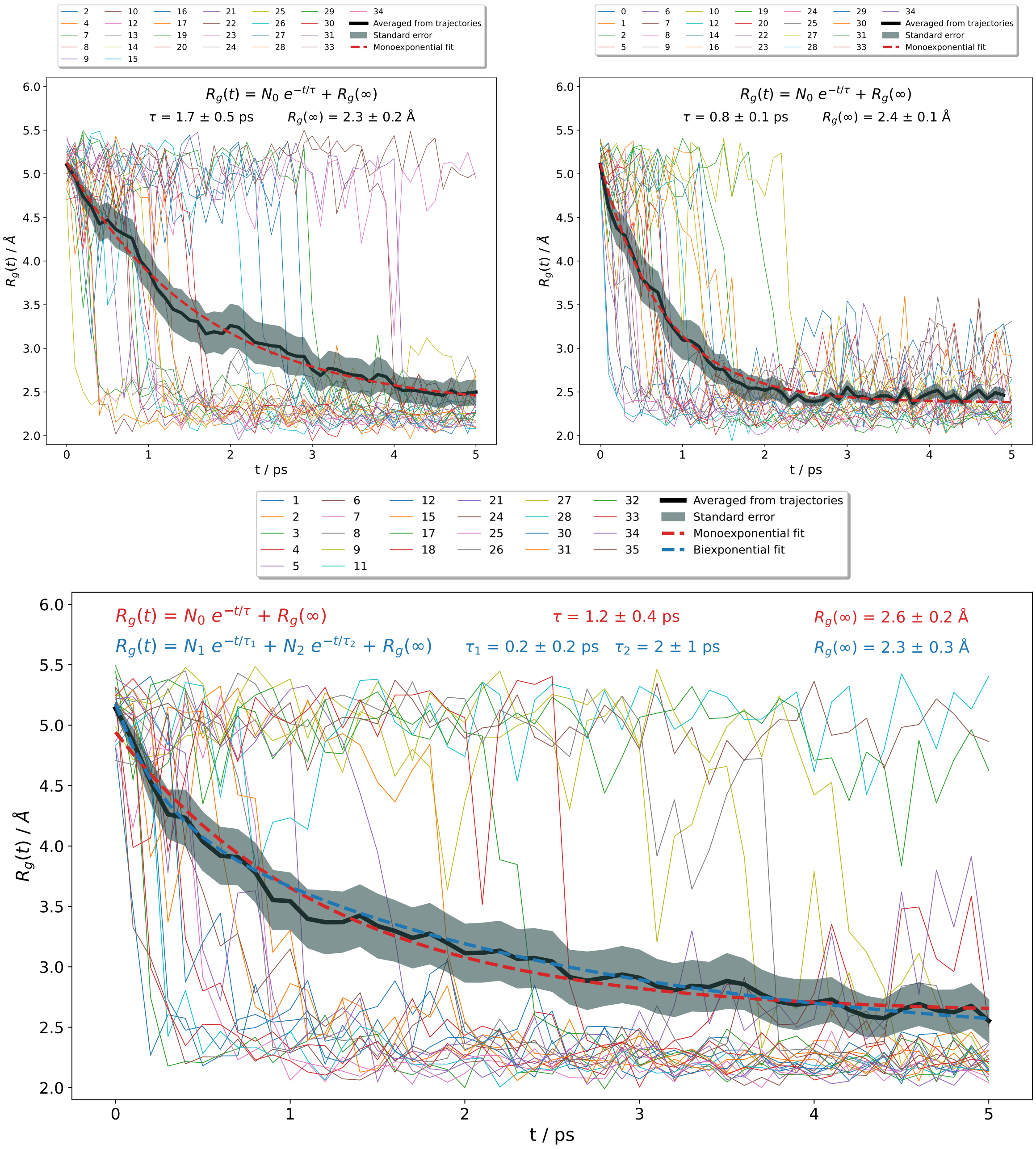

Figure S8: Gyration radius of the spin density as a function of time for the RPMD trajectories (colored thin lines) calculated at 300 K with a thermostat (top-left panel), 340 K with a thermostat (top-right panel), and 300 K with no thermostat (bottom panel). Black and red lines correspond to the mean gyration radius and its fit to a monoexponential decay law, respectively. The shaded area represents the standard error due to sampling. The results for the case of 300 K with no thermostat (bottom panel) also include a biexponential fit in blue.

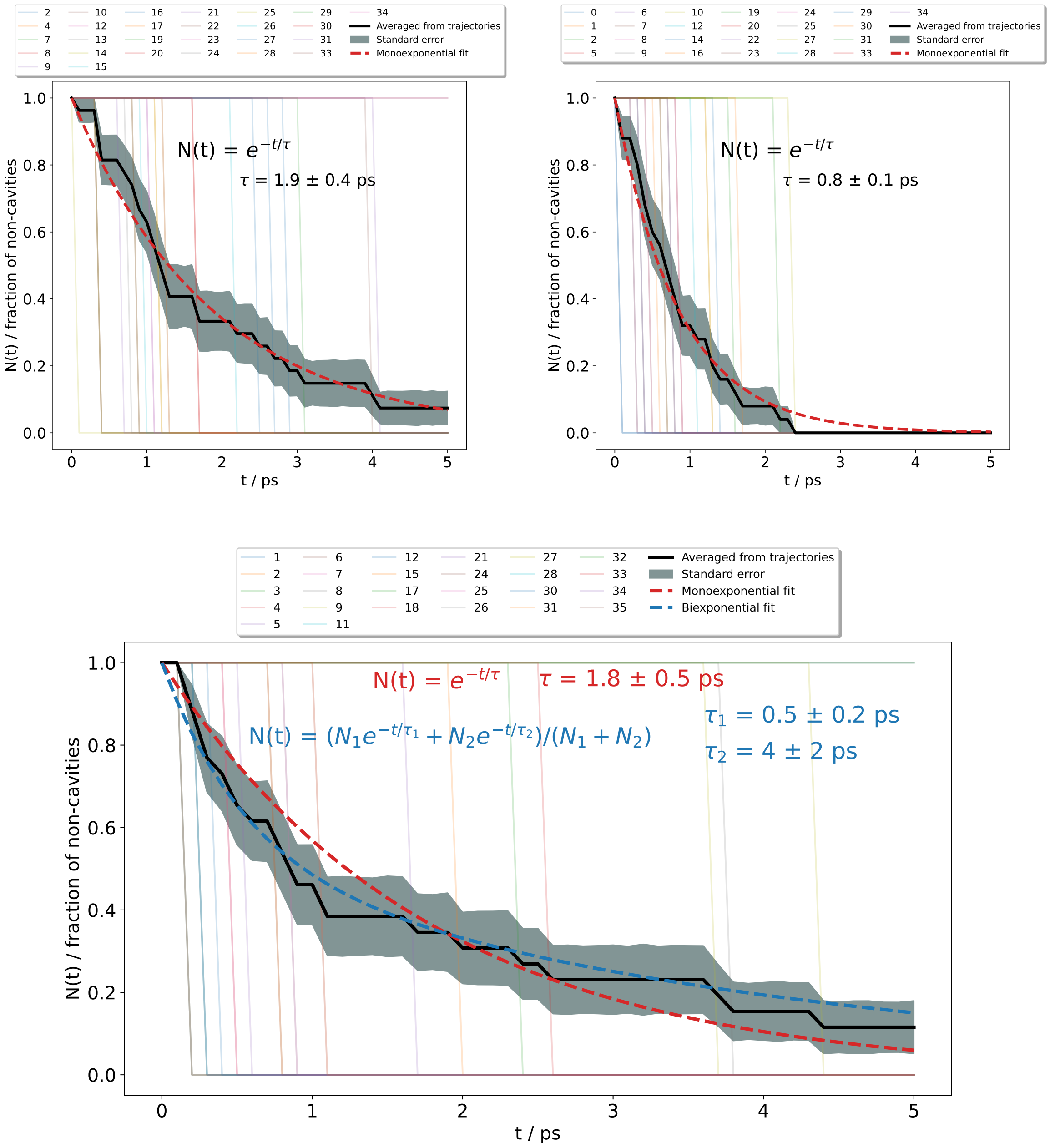

Figure S9: Ratio of trajectories (colored thin lines) that have not yet formed a cavity at any given time for the calculations at 300 K with a thermostat (top-left panel), 340 K with a thermostat (top-right panel), and 300 K with no thermostat (bottom panel, with two fits). Black and red lines correspond to the mean gyration radius and its fit to a monoexponential decay law, respectively. The shaded area represents the standard error due to sampling. The results for the case of 300 K with no thermostat (bottom panel) also include a biexponential fit in blue.

S-II.1.3 Hydrogen-Bond Network Analysis

For the analysis of the hydrogen-bond network, we used the tools available in the MDAnalysis package[14,15] for that purpose. The geometric criterion that we used to establish if a configuration corresponds to a hydrogen bond demanded a donor-hydrogen distance < 2.5 Å, a donor-acceptor distance < 3.5 Å, and a donor-hydrogen-acceptor angle $> 140°$.

S-II.2 Molecular Dynamics Simulations for H_2O, $OH\cdot$, and H_3O^+

To model all relevant species in the ionization process of liquid water, we have conducted *ab-initio* molecular dynamics simulations of solvated H_3O^+, $OH\cdot$, and bare liquid water using periodic boundary conditions and the PBE0+D3[16–18] functional as it demonstrated good results for bare liquid water.[19] The calculations mainly follow the setup described in Ref. 20. In short, we used the CP2K (version 9.1)[10,21,22] code and simulated 32 water molecules in a box of size $(9.865\,\text{Å})^3$ in an NVT ensemble with a time step of 0.5 fs using the PBE0+D3[16–18] functional. The cut-off radius for the interaction potential was set to 4.9 Å to avoid interaction with periodic copies of the simulation box. The double-zeta MOLOPT-SR-GTH[23] basis set together with the GTH-PBE pseudo potential was used. To keep the computational costs for exact exchange evaluation low, the auxiliary density matrix method (ADMM)[24] was employed using the auxiliary basis set `cFIT3`. The simulation temperature was 300 K and a Nose-Hoover thermostat[25–27] was used with a time constant of 10 fs for the first 5 ps and 50 fs afterward. The multi-grid parameters `NGRIDS=5`, `CUTOFF`=500.0 a.u., `REL_CUTOFF`=50.0 a.u. were used to have a sufficiently dense integration grid. To stabilize the evaluation of the exchange-correlation functional, additional smoothing of the electron density (`NN10_SMOOTH` and `NN10`) was used. For solvated H_3O^+ and $OH\cdot$ an analogous setup was used with an added proton for H_3O^+ and removed hydrogen atom for $OH\cdot$. For the latter, the respective unrestricted Kohn-Sham variant (UKS) was employed.

S-II.3 Calculation of X-ray Absorption Spectra

The calculation of x-ray absorption cross sections for energies above the ionization threshold is challenging, as it involves a description of electronic unbound states that have a continuous energy spectrum. The present calculations rely on Gaussian type orbitals, which can accurately represent bound states but conceptually fail for these unbound states. To nevertheless address absorption into electronic unbound states, we employ Gaussian basis sets generated employing the recipe of Kaufmann, Baumeister, and Jungen,[28] which locally adopts features of the electronic continuum and forms a so-called pseudo-continuum from which properties of the true continuum can be retrieved by the Stieltjes imaging procedure.[29]

Another challenge is the many-electron effects that appear due to the strong relaxation contribution that are involved when removing an electron from the core shell. We address this by making use of two different molecular orbital sets that are optimized for the initial and the core-ionized electronic configuration, respectively.

Assuming an average over orientations, the x-ray absorption cross section is

$$\sigma(E_{\text{photon}}) = 2\pi^2\alpha \sum_j f_{ij}\, \delta(\epsilon_{ij} - E_{\text{photon}}), \tag{4}$$

where f_{ij} is the oscillator strength for a transition from state i to state j, which is given in the dipole approximation as

$$f_{ij} = \frac{2}{3}\, \epsilon_{ij} \| \langle \psi_i | \mathbf{r} | \psi_j \rangle \|^2. \tag{5}$$

In Eqs. (4) and (5) $\epsilon_{ij} = E_j - E_i$ and $|\psi_i\rangle$, $|\psi_j\rangle$ are the initial and final, core-excited electronic states with energies E_i and E_j, respectively. We calculate the absorption process by employing individual (restricted open-shell) Hartree-Fock orbital optimizations for the initial state $|\psi_i\rangle$ with N electrons and the core-ionized state with $N-1$ electrons. For these Hartree-Fock calculations, we employ the 6-31++G(d) basis set and for the specific water molecule that absorbs the x-ray photon, we employ the larger 6-311++G(d) basis set. We further make use of localization procedures to make sure that all core orbitals are localized on

the respective oxygen atoms. The description of the final, core-excited electronic state $|\psi_j\rangle$ having N electrons is based on molecular orbitals optimized for the core-ionized $(N-1)$-electron configuration. These orbitals are further projected into a larger basis set where additional 11 s, 11 p, and 11 d-type Kaufmann basis functions[28] have been added on the respective core-ionized oxygen atom. The respective Kaufmann-basis functions are optimized for the specific ionized final charge state. For example, for the absorption by the hydrated hydronium cation (H_3O^+), Kaufmann-basis functions were taken that are generated for a dicationic continuum. Using this larger basis set, we describe the final electronic states $|\psi_j\rangle$ by diagonalizing the electronic Hamiltonian in the space of N-electron configurations, in which an electron has been added to a virtual or singly occupied orbital of the core-ionized configuration. For the cavity water molecules around a solvated electron, the final-state expansion is augmented by further electronic configurations where the solvated electron can occupy the n lowest unoccupied orbitals, where n is the number of water molecules included in the electronic structure calculation. This provides more flexibility in the final configuration expansion, accounting for the fact that the rather diffuse solvated electron orbital adapts quite significantly in the presence of the core hole and shows rather small overlap in the two orbital sets. For the calculations involving $OH\cdot$, configurational mixing in the two almost degenerate π hole orbitals is considered in the initial and final states as well.

The transition dipole operator in Eq. (5) is then calculated using Löwdin's generalized expression of transition matrices.[30] By taking two different sets of orbitals, orbital relaxation effects are directly taken into account and there is no imbalance by choosing a common orbital set adequate for a specific state (e.g., either the initial or the final state). The resulting absorption resonance energies ϵ_{ij} are thus considerably more accurate compared to resonance energies evaluated using a common orbital set. Compared to experimental data there remains an absolute energy shift of 1 eV–2 eV due to relativistic effects in the oxygen 1s shell (relativistic effects are about 0.8 eV for core ionization in neon[31]), limited basis set effects, and further many-electron correlation effects not considered in the calculation.

Equation (4) contains a δ function that reflects the energy conservation in the photoabsorption process. For the resonant absorption transitions that describe absorption into bound states, the x-ray absorption spectrum (XAS) is retrieved by replacing the δ function in Eq. (4) with a Gaussian function with a full width at half maximum of 1 eV. This way we incorporate fluctuations in the resonance position due to vibrations and finite-lifetime broadening.

The usage of spatially confined basis functions (here Gaussians) requires special treatment when approaching the electronic continuum, i.e., unbound electronic states. Instead of an explicit unbound electronic state with a continuous energy spectrum $|\psi(\epsilon)_j\rangle$, the calculation provides (if the basis set is sufficiently flexible) a pseudo-continuum variant of the final state $|\psi_j\rangle$ at discrete energy values E_j. This pseudo-continuum is artificial, but through the Stieltjes imaging procedure, one can approximately retrieve the cross sections or oscillator strengths for the true continuum. Essentially, the procedure relies on the fact that statistical moments of the pseudo-continuum oscillator strength f_{ij} represent the statistical moments obtained for the corresponding continuous oscillator strength $\tilde{f}_i(\epsilon)$, i.e.,

$$\int d\epsilon \, (\epsilon)^{-k} \tilde{f}_i(\epsilon) d\epsilon = \sum_j (\epsilon_{ij})^{-k} f_{ij}, \tag{6}$$

if the pseudo-continuum basis set is sufficiently flexible. For applying the Stieltjes imaging technique, we follow the prescription in Ref. 32. Specifically, we take into account negative moments up to the order of $k = 20$. Through the Stieltjes imaging procedure we obtain continuum-oscillator strengths $\tilde{f}_i(\epsilon)$ at discrete absorption energies ϵ that are eventually turned into a continuous function $\tilde{f}_i(\epsilon)$ via quadratic interpolation.

According to the Thomas-Reiche-Kuhn sum rule, the summed oscillator strength, which is the zeroth moment in Eq. (6), should fulfill

$$\sum_j f_{ij} = \int d\epsilon \, \tilde{f}_i(\epsilon) = 1. \tag{7}$$

To test our procedure, we inspected the summed oscillator strength for the considered absorption transitions and found values that are on average 0.93 (for H_2O), 0.95 (for $OH\cdot$), 1.01 (for H_2O neighboring H_3O^+), 1.25 (for H_3O^+), and 0.79 (for H_2O in a solvated electron cavity). The low value for a cavity water molecule is connected with the mentioned diffuse nature of the solvated electron that responds particularly sensitively to the creation of the core hole.

The two spectral parts, the bound-bound and the bound-continuum (obtained via the Stieltjes imaging procedure), are then merged at an energy value where both spectral parts overlap.

S-II.3.1 Sampling of structures for XAS calculations

From snapshots of the PBE0+D3 MD simulations, we calculated the XAS, for x-ray absorption of the OH radical, the H_3O^+ cation, water molecules neighboring H_3O^+, and water molecules in bulk water. To that end, the local environment of the absorbing species was considered in the calculation by incorporating the absorbing molecule and the closest water molecules in the electronic structure calculations for the x-ray absorption (QM region). The remaining water molecules up to a distance of 10.6 Å were incorporated as point charges, using values from the SPC/E water force field.[33] The selection was based on the oxygen-oxygen distance.

The individual criterion on how the geometrical structures for the QM region were selected is described in the following.

- Bulk water (MD): We picked snapshots of the water structure at two time frames, namely at 7.5 ps and at 10 ps of simulation time. From both snapshots, individual x-ray absorption spectra calculations were performed considering every water molecule as the absorbing water molecule. For each calculations the 9 closest water molecules were incorporated into the QM region.

- Bulk water (RPMD): We picked a single snapshot of the water structure (centroid po-

sitions) at 5 ps of simulation time of the RPMD trajectory. Otherwise, the calculation procedure was the same as for bulk water (MD).

- OH and H_3O^+: After 5 ps of equilibration time, we picked snapshots every 0.25 ps of the simulation box. From these snapshots, the absorbing OH / H_3O^+ molecule was identified by attributing each hydrogen atom to its closest oxygen atom and picking the oxygen atom that has been assigned one / three hydrogen atoms. Around the identified OH/H_3O^+ molecule, the 9 closest water molecules around the absorbing water molecule were considered in the electronic structure calculations (QM region).

- Water molecules next to H_3O^+: From the identified H_3O^+ molecules, the three closest water molecules were selected as the absorbing water molecules. For each of these water molecules, all water molecules were incorporated in the QM region that either have distance to H_3O^+ smaller than 3.5 Å or belong to the 9 closest water molecules around the absorbing water molecule.

- Water molecules next to e^-_{aq}: For the cavity surrounding the solvated electron, we used geometrical structures sampled from the neural-network-based RPMD simulations. We took snapshots of the centroid coordinates every 100 fs after cavity formation. To identify water molecules belonging to the cavity, we used the procedure described in Sec. S-II.1.1. In the calculation, we incorporated in the QM part all cavity water molecules plus those water molecules that are hydrogen-bonded to the respective absorbing water molecule and incorporated the remaining molecules up to a distance of 11.6 Å as point charges. The criterion used to select hydrogen-bonded molecules was a donor-acceptor distance < 3.17 Å and a donor-hydrogen-acceptor angle of $> 150°$.

The overall XAS difference spectrum was assembled by adding up the difference spectra for each species with respect to bulk water. To avoid any bias due to the difference between MD and RPMD sampling, we computed the cavity difference spectrum by subtracting from the calculated XAS of RPMD-cavity structures from those of RPMD-bulk-water structures.

S-III Additional Results

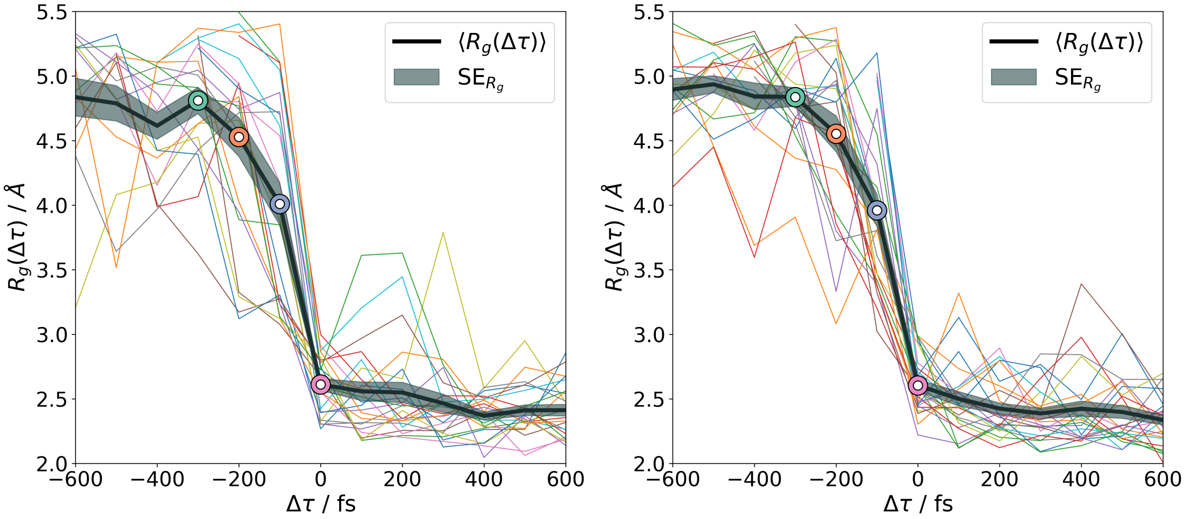

Figure S10: Evolution of the solvation process as a function of the relative formation time $\Delta\tau = t - \tau$, where τ represents the cavity-formation time of each trajectory. Gyration radius of the electron spin density, $R_g(\Delta\tau)$, for the various sampled trajectories for the simulations without a thermostat (left) and the simulations at 340 K.

For the simulations with thermostat and the simulations performed at higher temperature 340 K, we show in Fig. S10 the radius of gyrations $R_g(\Delta\tau)$ as a function of time relative to the solvation time (Equivalent results as in Fig. 4**A** in the main text). The figure demonstrates a very similar behavior for all three simulation setups. In addition to Fig. 4**A** in the main text, this further supports the idea that the collapse of the excess electron density is (apart from the onset) not influenced by fluctuations in the water structures.

References

(1) Ha, B.; DePonte, D. P.; Santiago, J. G., et al. Device design and flow scaling for liquid sheet jets. *Physical Review Fluids* **2018**, *3*, 114202.

(2) Crissman, C. J.; Mo, M.; Chen, Z.; Yang, J.; Huyke, D. A.; Glenzer, S. H.; Ledbetter, K.;

Nunes, J. P. F.; Ng, M. L.; Wang, H., et al. Sub-micron thick liquid sheets produced by isotropically etched glass nozzles. *Lab on a Chip* **2022**, *22*, 1365–1373.

(3) Droste, S.; Zohar, S.; Shen, L.; White, V. E.; Diaz-Jacobo, E.; Coffee, R. N.; Reid, A. H.; Tavella, F.; Minitti, M. P.; Turner, J. J., et al. High-sensitivity x-ray/optical cross-correlator for next generation free-electron lasers. *Optics Express* **2020**, *28*, 23545–23553.

(4) Heimann, P.; Reid, A.; Feng, Y.; Fritz, D. Fluorescence intensity monitors as intensity and beam-position diagnostics for X-ray free-electron lasers. *Journal of Synchrotron Radiation* **2019**, *26*, 358–362.

(5) Meibohm, J.; Schreck, S.; Wernet, P. Temperature dependent soft x-ray absorption spectroscopy of liquids. *Review of Scientific Instruments* **2014**, *85*, 103102.

(6) Li, J.; Nie, Z.; Zheng, Y. Y.; Dong, S.; Loh, Z.-H. Elementary electron and ion dynamics in ionized liquid water. *The Journal of Physical Chemistry Letters* **2013**, *4*, 3698–3703.

(7) Nagasaka, M.; Yuzawa, H.; Horigome, T.; Kosugi, N. Reliable absorbance measurement of liquid samples in soft X-ray absorption spectroscopy in transmission mode. *Journal of Electron Spectroscopy and Related Phenomena* **2018**, *224*, 93–99.

(8) Stenzel, O., et al. *The physics of thin film optical spectra*; Springer, 2015.

(9) Singraber, A.; Behler, J.; Dellago, C. Library-Based LAMMPS Implementation of High-Dimensional Neural Network Potentials. *Journal of Chemical Theory and Computation* **2019**, *15*, 1827–1840, DOI: 10.1021/acs.jctc.8b00770.

(10) Kühne, T. D.; Iannuzzi, M.; Del Ben, M.; Rybkin, V. V.; Seewald, P.; Stein, F.; Laino, T.; Khaliullin, R. Z.; Schütt, O.; Schiffmann, F.; Golze, D.; Wilhelm, J.; Chulkov, S.; Bani-Hashemian, M. H.; Weber, V.; Borštnik, U.; Taillefumier, M.;

Jakobovits, A. S.; Lazzaro, A.; Pabst, H.; Müller, T.; Schade, R.; Guidon, M.; Andermatt, S.; Holmberg, N.; Schenter, G. K.; Hehn, A.; Bussy, A.; Belleflamme, F.; Tabacchi, G.; Glöß, A.; Lass, M.; Bethune, I.; Mundy, C. J.; Plessl, C.; Watkins, M.; VandeVondele, J.; Krack, M.; Hutter, J. CP2K: An electronic structure and molecular dynamics software package - Quickstep: Efficient and accurate electronic structure calculations. *J. Chem. Phys.* **2020**, *152*, 194103, DOI: 10.1063/5.0007045.

(11) Lan, J.; Kapil, V.; Gasparotto, P.; Ceriotti, M.; Iannuzzi, M.; Rybkin, V. V. Simulating the ghost: quantum dynamics of the solvated electron. *Nature Communications* **2021**, *12*, 766, DOI: 10.1038/s41467-021-20914-0.

(12) Kapil, V.; Rossi, M.; Marsalek, O.; Petraglia, R.; Litman, Y.; Spura, T.; Cheng, B.; Cuzzocrea, A.; Meißner, R. H.; Wilkins, D. M.; Helfrecht, B. A.; Juda, P.; Bienvenue, S. P.; Fang, W.; Kessler, J.; Poltavsky, I.; Vandenbrande, S.; Wieme, J.; Corminboeuf, C.; Kühne, T. D.; Manolopoulos, D. E.; Markland, T. E.; Richardson, J. O.; Tkatchenko, A.; Tribello, G. A.; Speybroeck, V. V.; Ceriotti, M. i-PI 2.0: A universal force engine for advanced molecular simulations. *Computer Physics Communications* **2019**, *236*, 214–223, DOI: 10.1016/j.cpc.2018.09.020.

(13) Newville, M.; Stensitzki, T.; Allen, D. B.; Ingargiola, A. LMFIT: Non-Linear Least-Square Minimization and Curve-Fitting for Python. **2014**, DOI: 10.5281/ZENODO.11813.

(14) Gowers, R.; Linke, M.; Barnoud, J.; Reddy, T.; Melo, M.; Seyler, S.; Domański, J.; Dotson, D.; Buchoux, S.; Kenney, I.; Beckstein, O. MDAnalysis: A Python Package for the Rapid Analysis of Molecular Dynamics Simulations. 2016; pp 98–105, DOI: 10.25080/Majora-629e541a-00e.

(15) Michaud-Agrawal, N.; Denning, E. J.; Woolf, T. B.; Beckstein, O. MDAnalysis: A

toolkit for the analysis of molecular dynamics simulations. *Journal of Computational Chemistry* **2011**, *32*, 2319–2327, DOI: 10.1002/jcc.21787.

(16) Spencer, J.; Alavi, A. Efficient calculation of the exact exchange energy in periodic systems using a truncated Coulomb potential. *Phys. Rev. B* **2008**, *77*, 193110, DOI: 10.1103/PhysRevB.77.193110.

(17) Guidon, M.; Hutter, J.; VandeVondele, J. Robust Periodic Hartree-Fock Exchange for Large-Scale Simulations Using Gaussian Basis Sets. *J. Chem. Theory Comput.* **2009**, *5*, 3010–3021, DOI: 10.1021/ct900494g.

(18) Grimme, S.; Antony, J.; Ehrlich, S.; Krieg, H. A consistent and accurate ab initio parametrization of density functional dispersion correction (DFT-D) for the 94 elements H-Pu. *J. Chem. Phys.* **2010**, *132*, 154104, DOI: 10.1063/1.3382344.

(19) Todorova, T.; Seitsonen, A. P.; Hutter, J.; Kuo, I.-F. W.; Mundy, C. J. Molecular Dynamics Simulation of Liquid Water: Hybrid Density Functionals. *J. Phys. Chem. B* **2006**, *110*, 3685–3691, DOI: 10.1021/jp055127v.

(20) Apostolidou, C. OH radical in water from ab initio molecular dynamics simulation employing hybrid functionals. *The Journal of Chemical Physics* **2019**, *151*, 064111, DOI: 10.1063/1.5107479.

(21) VandeVondele, J.; Krack, M.; Mohamed, F.; Parrinello, M.; Chassaing, T.; Hutter, J. Quickstep: Fast and accurate density functional calculations using a mixed Gaussian and plane waves approach. *Computer Physics Communications* **2005**, *167*, 103–128, DOI: 10.1016/j.cpc.2004.12.014.

(22) VandeVondele, J.; Hutter, J. An efficient orbital transformation method for electronic structure calculations. *J. Chem. Phys.* **2003**, *118*, 4365–4369, DOI: 10.1063/1.1543154.

(23) VandeVondele, J.; Hutter, J. Gaussian basis sets for accurate calculations on molecular systems in gas and condensed phases. *J. Chem. Phys.* **2007**, *127*, 114105, DOI: 10.1063/1.2770708.

(24) Guidon, M.; Hutter, J.; VandeVondele, J. Auxiliary Density Matrix Methods for Hartree-Fock Exchange Calculations. *Journal of Chemical Theory and Computation* **2010**, *6*, 2348–2364, DOI: 10.1021/ct1002225, PMID: 26613491.

(25) Nosé, S. A unified formulation of the constant temperature molecular dynamics methods. *J. Chem. Phys.* **1984**, *81*, 511–519, DOI: 10.1063/1.447334.

(26) Nosé, S. A molecular dynamics method for simulations in the canonical ensemble. *Molecular Physics* **1984**, *52*, 255–268, DOI: 10.1080/00268978400101201.

(27) Martyna, G. J.; Klein, M. L.; Tuckerman, M. Nosé–Hoover chains: The canonical ensemble via continuous dynamics. *J. Chem. Phys.* **1992**, *97*, 2635–2643, DOI: 10.1063/1.463940.

(28) Kaufmann, K.; Baumeister, W.; Jungen, M. Universal Gaussian Basis Sets for an Optimum Representation of Rydberg and Continuum Wavefunctions. *J. Phys. B: At. Mol. Opt. Phys.* **1989**, *22*, 2223, DOI: 10.1088/0953-4075/22/14/007.

(29) Langhoff, P. W. Stieltjes Imaging of Atomic and Molecular Photoabsorption Profiles. *Chemical Physics Letters* **1973**, *22*, 60–64, DOI: 10.1016/0009-2614(73)80534-2.

(30) Löwdin, P.-O. Quantum Theory of Many-Particle Systems. I. Physical Interpretations by Means of Density Matrices, Natural Spin-Orbitals, and Convergence Problems in the Method of Configurational Interaction. *Phys. Rev.* **1955**, *97*, 1474–1489, DOI: 10.1103/PhysRev.97.1474.

(31) Niskanen, J.; Norman, P.; Aksela, H.; Ågren, H. Relativistic Contributions to Single

and Double Core Electron Ionization Energies of Noble Gases. *J. Chem. Phys.* **2011**, *135*, 054310, DOI: 10.1063/1.3621833.

(32) Müller-Plathe, F.; Diercksen, G. H. F. Perturbative-Polarization-Propagator Study of the Photoionization Cross Section of the Water Molecule. *Phys. Rev. A* **1989**, *40*, 696–711, DOI: 10.1103/PhysRevA.40.696.

(33) Berendsen, H. J. C.; Grigera, J. R.; Straatsma, T. P. The missing term in effective pair potentials. *J. Phys. Chem.* **1987**, *91*, 6269–6271, DOI: 10.1021/j100308a038.
